# The synergy between life’s essential 8 and muscle strength on cardiovascular disease risk

**DOI:** 10.3389/fmed.2025.1628066

**Published:** 2025-10-23

**Authors:** Linlong Li, Jianhong Xu, Li Huang, Xiangping Tu, Taiping Lin, Jirong Yue, Ning Ge, Chenkai Wu

**Affiliations:** ^1^Department of Geriatrics and National Clinical Research Center for Geriatrics, West China Hospital/West China School of Medicine, Sichuan University, Chengdu, Sichuan, China; ^2^Global Health Research Center, Duke Kunshan University, Kunshan, Jiangsu, China; ^3^Department of Experimental Medicine, West China Hospital/West China School of Medicine, Sichuan University, Chengdu, Sichuan, China

**Keywords:** muscle strength, life’s essential 8, cardiovascular disease, UK Biobank, interaction, precision prevention

## Abstract

**Background:**

While muscle strength is a known CVD risk factor in aging, its influence across the spectrum of Life’s Essential 8 (LE8) scores - the American Heart Association’s (AHA’s) comprehensive metric of cardiovascular health that includes diet, physical activity, sleep, nicotine exposure, body mass index, blood lipids, blood glucose, and blood pressure - remains unclear.

**Methods:**

We analyzed data from 237,682 individuals (mean age 55.64 ± 8.06 years) from UK Biobank. Muscle strength was assessed by grip strength and categorized into high, medium, and low based on tertiles. LE8 was classified into ideal (≥80), medium (50–79), and low (<50) based on AHA guideline. We used the Cox regression to examine the joint association of muscle strength and LE8 with incident CVD, including coronary heart disease, heart failure (HF) and stroke. Both multiplicative and additive interactions were examined.

**Results:**

Over a median 14.90-year follow-up, 26,159 incident CVD cases were recorded. Reduced muscle strength was not associated with CVD risk among persons with ideal LE8 (medium vs. high: HR = 0.91, 95% CI 0.79–1.05; low vs. high: HR = 1.03, 95% CI 0.90–1.17). However, a significant additive interaction was observed between low muscle strength and a low LE8 score, which accounted for 17% (AP = 0.17, 95% CI 0.10–0.24) of the excess CVD risk and corresponded to a threefold increased risk (HR = 3.03, 95% CI 2.72–3.37). This synergistic effect was particularly pronounced for HF, women, and younger individuals (<56 years). Among the individual LE8 components, blood glucose exhibited the strongest additive interaction with low muscle strength (RERI = 0.31, 95% CI 0.13–0.49; AP = 15, 95% CI 7–23%), followed by sleep, nicotine exposure, BMI, and physical activity.

**Conclusion:**

The cardiovascular risk associated with low muscle strength is contingent upon overall cardiovascular health. It poses a significant threat specifically when co-existing with poor cardiovascular health (a low LE8 score), highlighting the need for targeted interventions in this high-risk subgroup.

## 1 Introduction

Cardiovascular disease (CVD) is a leading cause of death worldwide, resulting in approximately 19.8 million deaths in 2022 (1). With changes in lifestyle and the increasing prevalence of chronic diseases such as diabetes and obesity, this burden is expected to rise further. Therefore, early management of CVD risk factors is crucial (2).

Muscle strength, an important biological indicator used to assess the intrinsic capabilities and functional status of older adults, is gradually becoming a key target for the prevention of CVD in an aging society (3). It is widely believed that a decline in muscle strength can influence the incidence of various CVD, such as coronary heart disease (CHD), heart failure (HF), and stroke (4). In addition to muscle strength, traditional risk factors, such as blood sugar, blood pressure, and blood lipids, are also considered significant risk factors for the incidence of CVD. Given the coexistence of these risk factors, the impact of muscle strength on CVD incidence may vary under different levels of management of these traditional risk factors (5). As the global population continues to age, the increased risk of CVD associated with age-related declines in muscle strength presents an even greater challenge to public health. Data indicate that by 2045, the number of individuals with low muscle strength in China alone could exceed 100 million (6). This trend portends substantial social and economic burdens, including reduced savings rates, labor shortages, delayed retirement ages, and strained public finances due to escalating age-related healthcare expenditures (7, 8). Consequently, investigating the differential effects of muscle strength on CVD incidence across varying levels of cardiovascular risk factor control is essential. Such research will refine the clinical precision of muscle-strength-targeted interventions and inform the development of more effective CVD prevention strategies, thereby optimizing public resource allocation and mitigating the clinical and societal challenges posed by resource scarcity in an aging population.

Life’s essential 8 (LE8), a concept initially introduced by the AHA in 2010 and subsequently updated in 2022, provides a standardized prevention framework for CVD and has serves as a vital assessment tool for managing individual CVD risk factors. The LE8 framework encompasses eight key components: four behavioral factors (diet, physical activity, sleep, and tobacco exposure) and four biological factors (BMI, blood lipid levels, blood pressure, and blood sugar) (9). Extensive research has consistently demonstrated that ideal LE8 is associated with significantly lower CVD risk, independent of age, socioeconomic status, or ethnicity (10–12). In fact, it is estimated that in the United States alone, ideal LE8 could prevent nearly 2 million new cases of CVD each year (13).

This study leverages data from the UK Biobank, utilizes LE8 metrics to comprehensively assess individual CVD risk management levels. We aim to investigate the joint and interactive associations of muscle strength and LE8 with incident CVD. Furthermore, we will analyze the interactions between each specific LE8 component (blood glucose, blood lipids, blood pressure, BMI, physical activity, diet, sleep, and nicotine exposure) and muscle strength on CVD risk. Specifically, we seek to elucidate how the association between muscle strength and CVD varies across different LE8 populations and to identify potential priorities for future interventions. By doing so, we hope to provide guidance for precise intervention strategies targeting muscle strength and to develop personalized approaches to mitigate the CVD risk associated with declines in muscle strength.

## 2 Methods

### 2.1 Study design and population

**Figure 1:**
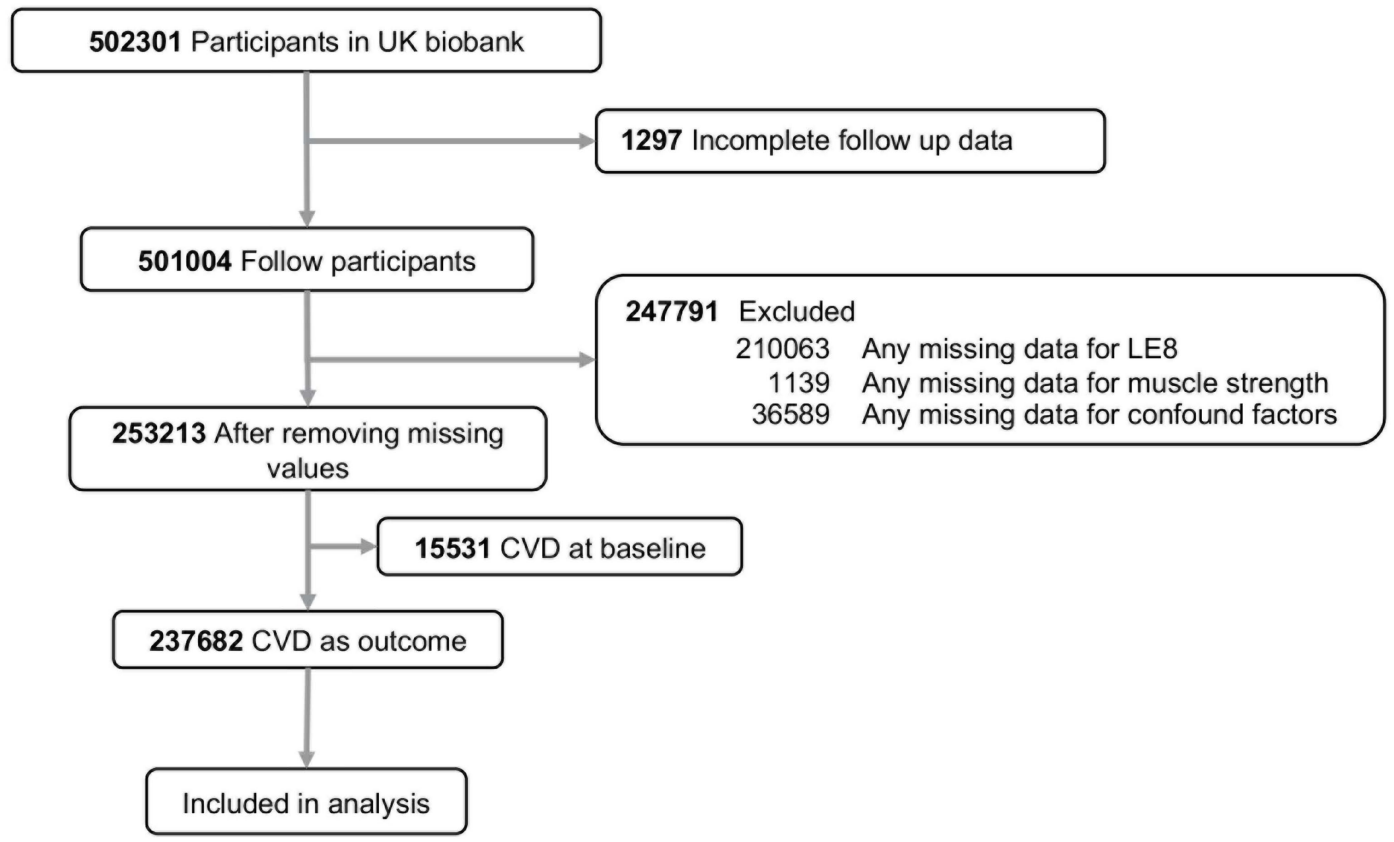
Flowchart of included participants. CVD, cardiovascular disease; LE8, life’s essential 8.

The UK Biobank recruited and followed over 500,000 participants aged 40–69 from 22 assessment centers across England, Scotland, and Wales between 2006 and 2010. All participants provided written informed consent at baseline recruitment, and the study was approved by the North West Multi-centre Research Ethics Committee and the Tulane University Biomedical Committee Institutional Review Board. For this analysis, we excluded individuals lost to follow-up (*n* = 1,297), those with missing data on LE8 (*n* = 210,063), muscle strength (*n* = 1,139), or key covariates (*n* = 36,589), and those with prevalent CVD at baseline (*n* = 15,531). The detail flow for the selection of the analyzed study sample in this study from the UK Biobank was provided in Figure 1.

### 2.2 Muscle strength

Muscle strength data were obtained from the UK Biobank, where it was assessed primarily through grip strength, a widely accepted and practical objective measure that serves as a reliable proxy for overall body muscle strength in large epidemiological cohorts (14–16). Grip strength was measured using a Jamar J00105 hydraulic dynamometer, which assesses isometric strength (without movement) and is adjustable to accommodate hand sizes in 5.5-inch increments. The device provides a dual-scale readout of grip strength ranging from 0 to 90 kilograms, and a “Peak grip strength” pin remains in place once the grip is released to record the maximum value. The dynamometer was calibrated before each use to ensure accuracy. Participants were seated upright in a chair with their forearms resting on the armrests. While holding the dynamometer, participants kept their elbows close to their bodies, bent at a 90-degree angle, with their forearms pointing forward and their thumbs positioned on top. Wrists were kept straight, and hands were either pointed forward or slightly outward. Participants were instructed to squeeze the dynamometer handle as forcefully as possible for approximately 3 s, with verbal encouragement provided. Measurements were taken for both the right and left hands. For subsequent analyses, the mean muscle strength from both hands was calculated and expressed in kilograms. Following prior studies, muscle strength was categorized into three groups—high, medium, and low—based on trichotomies stratified by gender and age (Supplementary Table 1; Supplementary Figure 1) (17–19).

### 2.3 LE8

LE8 was assessed using the LE8 score, which comprises eight components: (1) diet, (2) physical activity, (3) tobacco/nicotine exposure, (4) sleep, (5) BMI, (6) non–high-density lipoprotein (non-HDL) cholesterol, (7) blood glucose, and (8) blood pressure. Dietary scores were constructed based on AHA guidelines and adapted to align with UK Biobank data (20–23). Self-reported information was collected on physical activity (minutes of moderate to vigorous activity per week), tobacco/nicotine exposure (use of combustible tobacco and exposure to secondhand smoke), sleep duration (hours per night), and medication use during participant interviews. Height, weight, and blood pressure were measured at the assessment centers. BMI was calculated as weight in kilograms divided by height in meters squared. Blood pressure was measured twice by a trained nurse, and the mean systolic and diastolic values were used for analysis. Automated blood pressure measurements were preferred; however, manual measurements were used if automated readings were unavailable. Laboratory measurements of serum cholesterol and glycated hemoglobin (HbA1c) were performed at a central laboratory. Serum cholesterol was measured using an enzymatic method, and non-HDL cholesterol was calculated by subtracting HDL cholesterol from total cholesterol. HbA1c levels were determined using high-performance liquid chromatography. Detailed information and scoring algorithms for each LE8 component were in Supplementary Tables 2, 3. Each LE8 metric was scored on a scale of 0 to 100 points. The overall LE8 score was calculated by averaging the scores of the eight components, yielding a total score ranging from 0 to 100, with higher scores indicating better LE8. Based on AHA guidelines, LE8 was categorized as low (score <50), medium (score 50–79), or ideal (score ≥80).

### 2.4 Outcome

In the UK Biobank study, participant survival status was primarily determined through linkage to hospital admission records and death registration data. Detailed information on the linkage process is available online. The outcome of this study was the incidence of CVD, which included three major components: CHD, HF, and stroke. Outcomes were defined according to the ICD-10: CHD was coded as I20-I25, HF as I50, and stroke as I60-I64 (Supplementary Table 4).

### 2.5 Covariates

Covariates including age, gender, race, height, education, annual household income, and alcohol intake were assessed through a touchscreen questionnaire. Information on hyperthyroidism, depression, and chronic kidney failure (CKF) was obtained from hospital admission records and death registration data. Educational attainment was classified into seven categories: “College or University degree” was considered a university-level education, “A levels/AS levels or equivalent” as a high school education, and “O levels/GCSEs or equivalent,” “CSE or equivalent,” “NVQ or HND or HNC or equivalent,” “Other professional qualifications (e.g., nursing, teaching),” and “None of the above” were grouped as other qualifications. Annual household income before taxes was categorized into four groups: (1) less than £18,000, (2) £18,000 to £29,999, (3) £30,000 to £51,999, and (4) more than £52,000. Alcohol intake was categorized into six frequency groups: (1) Never, (2) Special occasions only, (3) 1–3 times per month, (4) 1–2 times per week, (5) 3–4 times per week, and (6) Daily or almost daily. Hyperthyroidism, depression, and CKF were identified as present at baseline based on diagnosis timing from hospital records. Further details regarding covariate collection can be found on the UK Biobank website.

### 2.6 Statistical analysis

Baseline characteristics of the study sample were summarized as percentages for categorical variables and as means with standard deviations for continuous variables, stratified by gender. Comparisons between categorical variables were conducted using chi-square tests, while t tests were employed for continuous variables.

Participants were categorized into nine groups based on muscle strength and LE8, with high muscle strength and ideal LE8 serving as the reference group. Cox proportional hazards models were used to assess the risk of incident CVD across these groups, using follow-up time as the time scale. Models were adjusted for pre-specified covariates, including: (1) demographic factors: age (continuous), sex (male/female), race (white/other), and height (continuous); (2) socioeconomic factors: education level (college, high school, or other), annual household income (categorized into four groups), and drinking frequency (six groups); and (3) comorbidities: history of hyperthyroidism, depression, and chronic renal failure, obtained from baseline hospital records (yes/no).

To assess the potential interaction between muscle strength and LE8 on the incidence of CVD, both multiplicative and additive interaction effects were evaluated. Multiplicative interaction was assessed by incorporating product terms into the Cox models. Additive interaction was evaluated by the relative excess risk due to interaction (RERI), attributable proportion (AP), and synergy index (SI). To identify which LE8 component contributed most to the association, we compared the magnitude of interaction effects between low muscle strength and low LE8 components, including diet, physical activity, nicotine exposure, sleep, BMI, blood lipids, blood glucose, and blood pressure.

Several sensitivity analyses were conducted: (1) The dose–response relationship between the continuous LE8 score and CVD risk was examined using restricted cubic splines. If the relationship was linear, we estimated the hazard ratio (HR) for a 10-point decrease in LE8 score within each muscle strength stratum. (2) The analysis was repeated using relative grip strength (absolute grip strength / BMI) instead of absolute strength. (3) A competing risk analysis was performed using a cause-specific hazards model, treating all-cause mortality as a competing event. (4) To address potential reverse causality, we excluded the first 3 years of follow-up. (5) Stratified analyses were conducted by sex (males and females), age group (<56, 56–65, >65 years), and CVD subtype (CHD, HF, stroke).

All statistical analyses were conducted using R version 4.4.3 (R Foundation for Statistical Computing, Vienna, Austria). Two-sided *p*-values < 0.05 were considered statistically significant.

## 3 Results

### 3.1 Baseline characteristics

Table 1 summarized the baseline characteristics of participants. A total of 237,682 participants were analyzed, including 111,646 men and 126,036 women. The mean (SD) value of the age was 55.64 (8.06). Compared to women, men had higher muscle strength and physical activity scores but lower scores for diet, nicotine exposure, sleep, BMI, lipids, glucose, blood pressure, and overall LE8 scores. Notably, neither men nor women achieved ideal LE8, with particularly low scores observed for blood pressure and lipids, while glucose scores were relatively higher. Over a median follow-up of 14.90 years (interquartile range [IQR], 14.07–15.59 years), corresponding to 3,386,051 person-years, 26,159 CVD events were recorded. The incidence of CVD was higher in men (14.74%) compared to women (7.69%).

**Table 1:** Baseline characteristics of study participants with CVD as outcome.

Characteristics	Total	Male	Female	*p*-value
No. of participants	237,682	111,646	126,036	
Age, mean (SD)	55.64 (8.06)	55.91 (8.18)	55.41 (7.95)	*p* < 0.001
Race:				*p* = 0.003
White, *n* (%)	227,785 (95.84)	107,144 (95.97)	120,641 (95.72)	
Others, *n* (%)	9,897 (4.16)	4,502 (4.03)	5,395 (4.28)	
Height, mean (SD)	169.12 (9.27)	176.16 (6.74)	162.88 (6.23)	*p* < 0.001
Education:				*p* < 0.001
College or university degree, *n* (%)	86,787 (36.51)	42,310 (37.90)	44,477 (35.29)	
High school education, *n* (%)	29,086 (12.24)	12,524 (11.22)	16,562 (13.14)	
Others, *n* (%)	121,809 (51.25)	56,812 (50.88)	64,997 (51.57)	
Income:				*p* < 0.001
Less than 18,000, *n* (%)	47,230 (19.87)	18,962 (16.98)	28,268 (22.43)	
18,000 to 30,999, *n* (%)	59,093 (24.86)	26,201 (23.47)	32,892 (26.10)	
31,000 to 51,999, *n* (%)	64,810 (27.27)	31,466 (28.18)	33,344 (26.46)	
Greater than 52,000, *n* (%)	66,549 (28.00)	35,017 (31.37)	31,532 (25.01)	
Alcohol:				*p* < 0.001
Never, *n* (%)	16,057 (6.76)	5,841 (5.23)	10,216 (8.11)	
Special occasions only, *n* (%)	24,830 (10.45)	7,213 (6.46)	17,617 (13.98)	
1–3 times per month, *n* (%)	26,786 (11.27)	9,910 (8.88)	16,876 (13.39)	
1–2 times per week, *n* (%)	61,350 (25.81)	28,580 (25.60)	32,770 (26.00)	
3–4 times per week, *n* (%)	57,192 (24.06)	30,261 (27.10)	26,931 (21.37)	
Daily or almost daily, *n* (%)	51,467 (21.65)	29,841 (26.73)	21,626 (17.15)	
Hyperthyroidism, *n* (%)	2,414 (1.02)	470 (0.42)	1944 (1.54)	*p* < 0.001
Depression, *n* (%)	18,892 (7.95)	6,484 (5.81)	12,408 (9.84)	*p* < 0.001
CKF, *n* (%)	2,111 (0.89)	980 (0.88)	1,131 (0.90)	*p* = 0.63
Muscle strength, mean (SD)	31.46 (11.03)	40.11 (8.65)	23.79 (6.14)	*p* < 0.001
Diet score, mean (SD)	30.26 (31.03)	26.49 (31.38)	33.60 (30.52)	*p* < 0.001
Physical activity score, mean (SD)	73.92 (37.86)	75.23 (37.18)	72.75 (38.42)	*p* < 0.001
Nicotine exposure score, mean (SD)	79.23 (32.76)	76.65 (34.14)	81.51 (31.31)	*p* < 0.001
Sleep score, mean (SD)	89.90 (18.00)	89.88 (17.80)	89.91 (18.16)	*p* = 0.66
BMI score, mean (SD)	69.72 (28.18)	67.23 (26.53)	71.92 (29.38)	*p* < 0.001
Lipid score, mean (SD)	47.30 (29.18)	45.95 (28.32)	48.49 (29.86)	*p* < 0.001
Glucose score, mean (SD)	91.80 (18.73)	90.90 (20.01)	92.59 (17.48)	*p* < 0.001
Blood pressure score, mean (SD)	36.74 (34.97)	30.73 (31.02)	42.06 (37.32)	*p* < 0.001
LE8, mean (SD)	64.86 (12.56)	62.88 (12.07)	66.60 (12.73)	*p* < 0.001
CVD, *n* (%)	26,159 (11.01)	16,462 (14.74)	9,697 (7.69)	*p* < 0.001

Data are represented as mean (standard deviation, SD) or number (%). CKF, chronic kidney failure; LE8, life’s essential 8; CVD, cardiovascular disease.

### 3.2 Joint association of muscle strength and LE8 with CVD

**Figure 2:**
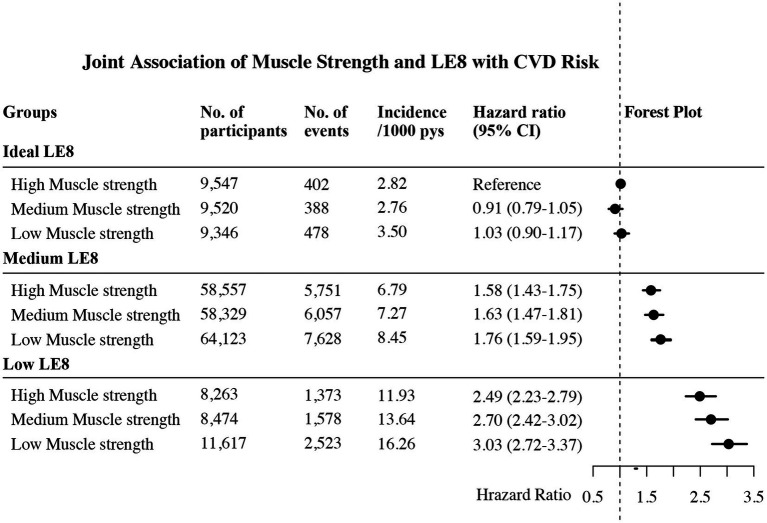
Joint association of muscle strength and LE8 on the risk of CVD. LE8 was categorized as low (LE8 score <50), medium (LE8 score 50–79), or ideal (LE8 score ≥80) based on AHA guidelines. Muscle strength was categorized into three groups—high, medium, and low—based on trichotomies stratified by gender and age. Multivariable adjusted for age, sex, race, height, education, income, alcohol, hyperthyroidism, depression and chronic kidney failure. No., number; CVD, cardiovascular disease; LE8, life’s essential 8; pys, person years.

Figure 2 presented the combined effect of muscle strength and LE8 on the risk of incident CVD. Among individuals with ideal LE8, a reduction in muscle strength, in isolation, did not significantly elevate the risk of CVD incidence, as the HRs for medium and low muscle strength in individuals with ideal LE8 were 0.91 (95% CI, 0.79–1.05) and 1.03 (95% CI, 0.90–1.17), respectively. In the medium LE8 group, we recorded HRs of 1.58 (95% CI, 1.43–1.75), 1.63 (95% CI, 1.47–1.81), and 1.76 (95% CI, 1.59–1.95) for the risk of CVD incidence in the high, medium, and low muscle strength groups, respectively. Notably, among all groups, low LE8 combined with low muscle strength conferred the highest CVD risk, where we could observe a staggering 203% increase in CVD risk.

Sensitivity analyses confirmed the robustness of these findings. The relationship between LE8 and CVD risk was linear (P for non-linearity = 0.311); accordingly, a 10-point decrease in the LE8 score was associated with an approximately 30% higher CVD risk across all muscle strength strata (Supplementary Figure 2, Supplementary Table 5). Other sensitivity analyses—including the use of relative grip strength, exclusion of the first 3 years of follow-up, adjustment for competing risks, and stratification by sex, age, and CVD subtype—consistently confirmed our primary findings: isolated low muscle strength within the ideal LE8 group was not associated with significantly increased CVD risk, whereas the combination of low muscle strength and low LE8 substantially elevated risk, particularly among women (268% increase), younger participants (387% increase), and HF patients (343% increase) (Supplementary Tables 6–13).

### 3.3 Interactive association of muscle strength and LE8 with CVD

Table 2 demonstrated the interaction effects between muscle strength and LE8. The multiplicative interaction between low muscle strength and medium LE8 was significant (HR = 1.19, 95% CI: 1.02–1.39). On an additive scale, this combination yielded a relative excess risk due to interaction (RERI) of 0.30 (95% CI: 0.08–0.52) and an attributable proportion (AP) of 11% (95% CI: 3–19%). For the combination of low muscle strength and low LE8, the multiplicative interaction was also significant (HR = 1.19, 95% CI: 1.02–1.38). The corresponding additive interaction was stronger, with a RERI of 0.52 (95% CI: 0.31–0.73), an AP of 17% (95% CI: 10–24%), and a synergy index (SI) of 1.34 (95% CI: 1.16–1.55).

**Table 2:** Interaction effect between muscle strength and LE8 on the incidence of CVD.

Muscle strength	LE8	Multiplicative interaction	Additive interaction		
			RERI	AP	SI
Medium	Medium	1.13 (0.98–1.31)	0.14 (0.005–0.28)	0.09 (−0.001–0.18)	–
	Low	1.09 (0.95–1.25)	0.16 (0.03–0.30)	0.09 (0.01–0.17)	1.27 (0.98–1.65)
Low	Medium	1.19 (1.02–1.39)	0.30 (0.08–0.52)	0.11 (0.03–0.19)	–
	Low	1.19 (1.02–1.38)	0.52 (0.31–0.73)	0.17 (0.10–0.24)	1.34 (1.16–1.55)

LE8 was categorized as low (LE8 score <50), medium (LE8 score 50–79), or ideal (LE8 score ≥80) based on AHA guidelines. Muscle strength was categorized into three groups-high, medium, and low-based on trichotomies stratified by gender and age. Analysis was adjusted for age, sex, race, height, education, income, alcohol, hyperthyroidism, depression and chronic kidney failure. LE8, Life’s essential 8; RERI, relative excess risk due to interaction; AP, attributable proportion; SI, synergy index.

Sensitivity analyses employing relative grip strength, exclusion of the first three follow-up years, and stratification by sex, age, and CVD subtype consistently demonstrated an additive interaction between low muscle strength and low LE8. This interaction was particularly pronounced in women (RERI = 0.60), individuals aged <56 years (RERI = 1.15), and HF patients (RERI = 0.89) (Supplementary Tables 6–8, 10–13).

### 3.4 Interactive association of muscle strength and individual LE8 component with CVD

Table 3 presents the multiplicative and additive interactions between low muscle strength and low levels of each individual LE8 component. The strength of the additive interaction varied across components. On the additive scale, significant interactions were observed for blood glucose (RERI = 0.31, 95% CI: 0.13–0.49; AP = 15, 95% CI: 7–23%), sleep (RERI = 0.20, 95% CI: 0.05–0.35; AP = 13, 95% CI: 4–22%), nicotine exposure (RERI = 0.19, 95% CI: 0.06–0.32; AP = 10, 95% CI: 3–16%), BMI (RERI = 0.18, 95% CI: 0.06–0.29; AP = 9, 95% CI: 3–14%), and physical activity (RERI = 0.09, 95% CI: 0.01–0.17; AP = 7, 95% CI: 1–13%). On the multiplicative scale, significant interactions were observed for blood glucose (HR = 1.14, 95% CI: 1.02–1.26) and sleep (HR = 1.12, 95% CI: 1.002–1.25).

**Table 3:** Interaction effects between low muscle strength and individual low LE8 scores for CVD incidence.

LE8 Categories	Multiplicative interaction	Additive interaction		
		RERI	AP	SI
Glucose	1.14 (1.02–1.26)	0.31 (0.13–0.49)	0.15 (0.07–0.23)	1.41 (1.14–1.74)
Sleep	1.12 (1.002–1.25)	0.20 (0.05–0.35)	0.13 (0.04–0.22)	1.52 (1.08–2.15)
Nicotine exposure	1.06 (0.97–1.15)	0.19 (0.06–0.32)	0.10 (0.03–0.16)	1.25 (1.06–1.48)
BMI	1.03 (0.95–1.12)	0.18 (0.06–0.29)	0.09 (0.03–0.14)	1.20 (1.06–1.37)
Physical activity	1.06 (0.99–1.14)	0.09 (0.01–0.17)	0.07 (0.01–0.13)	1.38 (1.001–1.91)
Diet	1.03 (0.95–1.11)	0.05 (−0.04–0.13)	0.04 (−0.03–0.10)	1.19 (0.83–1.72)
Blood pressure	0.94 (0.83–1.07)	0.03 (−0.11–0.17)	0.01 (−0.06–0.09)	1.03 (0.87–1.22)
Lipid	0.81 (0.75–0.88)	−0.24 (−0.34 – –0.14)	−0.18 (−0.26 – –0.11)	0.54 (0.46–0.64)

LE8 was categorized as low (LE8 score <50), medium (LE8 score 50–79), or ideal (LE8 score ≥80) based on AHA guidelines. Muscle strength was categorized into three groups-high, medium, and low-based on trichotomies stratified by gender and age. Analysis was adjusted for age, sex, race, height, education, income, alcohol, hyperthyroidism, depression and chronic kidney failure. LE8, Life’s essential 8; BMI, body mass index; RERI, relative excess risk due to interaction; AP, attributable proportion; SI, synergy index.

## 4 Discussion

This large-scale prospective study utilized data from the UK Biobank to investigate the interactive and joint associations between muscle strength, LE8, and CVD incidence. Our findings demonstrate that decline in muscle strength alone does not lead to a statistically significant increase in CVD risk when ideal LE8 is maintained. Specifically, in populations with ideal LE8, the HR for CVD incidence in medium and low muscle strength groups were 0.91 (95% CI, 0.79–1.05) and 1.03 (95% CI, 0.90–1.17), respectively, compared to the high muscle strength group. In contrast, a pronounced additive interaction was observed when low muscle strength coexisted with a low LE8 score, accounting for 17% of the excess CVD risk and corresponding to a threefold increase in risk. Sensitivity analyses further revealed that this additive interaction was particularly prominent for HF, elderly, and female participants. Furthermore, assessment of individual LE8 components revealed that the additive interaction with low muscle strength was strongest for blood glucose, followed by sleep, nicotine exposure, BMI, and physical activity. These findings suggest that changes in muscle strength may exert different biological effects on the CVD incidence at varying levels of LE8, providing important public health evidence to support the development of targeted interventions aimed at reducing muscle strength-related CVD incidence.

Unlike previous studies, our research found that a decline in muscle strength does not lead to a statistically significant increase in the risk of CVD when ideal LE8 is maintained. Many earlier studies have simplistically linked reduced muscle strength to an increased risk of CVD, often overlooking the biological roles of other traditional risk factors (24, 25). For instance, a study utilizing data from the China Health and Retirement Longitudinal Study (CHARLS), which included 9,369 participants followed for 3 years, suggested that optimal muscle strength could reduce CVD risk by approximately 10% (24). Similarly, a large prospective cohort study in Sweden indicated that higher muscle strength provides protective benefits against various CVD subtypes, including ischemic heart disease, heart failure, and stroke (25). In contrast, our research employed the LE8 metrics defined by the AHA, a widely recognized and standardized indicator that encompasses both biological factors (such as blood glucose, lipids, blood pressure, and obesity) and lifestyle factors (including diet, sleep, smoking, and physical activity), revealing that in populations with ideal LE8, a decline in muscle strength alone does not significantly increase CVD risk (9). This novel insight, which has been largely overlooked in prior research, highlights the importance of considering LE8 levels when assessing the impact of muscle strength on CVD risk. Future analyses should incorporate LE8 metrics to more accurately evaluate the changes in CVD risk associated with muscle strength across different populations.

Several potential mechanisms can explain why the decline in muscle strength does not lead to an increased risk of CVD in populations with ideal LE8. Firstly, the reduction in muscle strength may be offset or reversed by the maintenance of ideal LE8, as lifestyle factors such as physical activity, dietary quality, and metabolic health are closely related to muscle function (26). Secondly, the increased risk of CVD associated with declining muscle strength may be counterbalanced by ideal LE8 (22). Thirdly, adverse pathophysiological changes associated with CVD—such as chronic inflammation, oxidative stress, mitochondrial dysfunction, and lipid metabolism disorders—resulting from the decline in muscle strength, may be corrected by maintaining ideal LE8. Previous studies have shown that adherence to ideal LE8 is associated with lower systemic inflammation, improved lipid profiles, enhanced mitochondrial function, and reduced reactive oxygen species production, all of which are related to the mechanisms of muscle strength decline and CVD (27–29).

Conversely, a significant additive interaction was observed when low muscle strength co-occurred with a low LE8 score. This interaction accounted for approximately 17% of the excess CVD risk in this group, which corresponded to a threefold increase in risk. Stratified analyses further demonstrated that this synergistic effect was especially prominent for HF, younger participants (<56 years), and women, accounting for approximately 20, 24, and 16% of excess risk, and corresponding to 343, 387, and 268% increases in risk, respectively. These findings suggest that the impact of muscle strength on CVD risk is interdependent with LE8 status, rather than being independent. Previous research has similarly indicated that ideal LE8 can mitigate the CVD risk associated with low muscle strength by nearly 90% (30). Individuals with low muscle strength are also more susceptible to metabolic disorders such as dyslipidemia, obesity, impaired glucose metabolism, and hypertension—all of which contribute to poor LE8 (3). Given the global trends of aging and lifestyle changes, the substantial number of additional CVD cases attributable to this additive interaction carries significant public health implications. Future clinical interventions aimed at enhancing muscle strength should particularly focus on populations with low LE8, especially younger adults, women, and HF patients. For these high-risk groups, tailored resistance training programs—emphasizing progressive overload, social support, and professional supervision—are recommended to ensure safety and adherence. Community-based initiatives that integrate structured exercise, social interaction, and ability-appropriate challenges may offer a particularly effective implementation model (31). To establish robust evidence for these strategies, future research should prioritize randomized controlled trials and longitudinal studies evaluating the efficacy of such integrated interventions in reducing CVD incidence among high-risk individuals.

The strength of the additive interaction with low muscle strength varied considerably across individual LE8 components. Our analysis identified blood glucose as the component with the strongest interaction, followed by sleep health, nicotine exposure, BMI, and physical activity. This hierarchy suggests that interventions aimed at preserving muscle strength could yield the greatest benefit if prioritized for individuals with concurrent dysglycemia, poor sleep health, smoking habits, elevated BMI, or physical inactivity, thereby optimizing resource allocation for CVD prevention. An unexpected finding was a negative additive interaction between low muscle strength and dyslipidemia. We propose two potential explanations for this observation. First, patients with dyslipidemia are typically on long-term lipid-lowering therapy (e.g., statins) (32). While primarily prescribed for lipid control, statins may exert pleiotropic effects that potentially attenuate age-related muscle decline, thereby counteracting the expected risk from low muscle strength (33–36). Second, the standardized deduction for lipid-lowering medication use in the LE8 scoring algorithm, while necessary for uniformity, may not fully account for the heterogeneous effects of different drug regimens (e.g., monotherapy versus combination therapy), potentially introducing residual confounding (9, 32). Despite this observation, the AHA’s LE8 framework remains a robust and authoritative tool. Future studies are warranted to dissect the complex interplay between lipid management, muscle strength, and CVD risk.

The strengths of this study lie in its large sample size, the objective quantification of CVD risk through LE8, and the application of rigorous statistical methods to investigate the interactive and joint effects of muscle strength, LE8, and CVD incidence. Our research suggests that the biological effects of muscle strength on CVD incidence vary significantly across different LE8 populations. Consequently, future health education and clinical initiatives targeting muscle strength should be prioritized for populations with low LE8 scores, particularly those with poor glucose control, sleep disorders, nicotine exposure, obesity, or physical inactivity, to enable precise prevention in aging societies.

However, several limitations should be acknowledged. First, muscle strength was classified using a simplified three-tier system rather than a continuous scoring system, which may limit the granularity of our analysis. Nevertheless, the three-tier classification is widely used in large-scale epidemiological studies and remains an effective approach for population-level research. Second, LE8 was assessed only at baseline, and potential longitudinal changes in LE8 status and muscle strength were not accounted for. Future studies should incorporate repeated measures of LE8 and muscle strength to better capture their dynamic interactions. Third, the UK Biobank cohort primarily consists of White European individuals, which limits the generalizability of our findings to other racial and ethnic groups. Fourth, the UK Biobank population may exhibit healthier behaviors than the general population, which could limit the representativeness of the study. However, representativeness is not a prerequisite for valid exposure-disease relationships (37). Fifth, despite standardized protocols and training, the involvement of multiple assessors across centers could introduce measurement variability, though this is mitigated by the large sample size and stringent quality control. Sixth, residual confounding from unmeasured (e.g., genetic factors) or imperfectly measured variables cannot be excluded. Lastly, most participants were aged 40–70 years, so further research is required to assess the applicability of these findings to other age groups.

## 5 Conclusion

In conclusion, our large prospective cohort study demonstrates that the association between muscle strength and incident CVD is critically dependent on an individual’s overall cardiovascular health, as measured by the LE8 score. This relationship is highlighted by the stark contrast in risk across LE8 strata: within the ideal LE8 group, diminished muscle strength was not associated with a significant increase in CVD risk (medium vs. high: HR = 0.91, 95% CI 0.79–1.05; low vs. high: HR = 1.03, 95% CI 0.90–1.17). In marked contrast, the confluence of low muscle strength and a low LE8 score exhibited a potent synergistic effect, corresponding to a threefold increase in CVD risk, with 17% of this excess risk attributable to their additive interaction.

These findings reframe the risk of low muscle strength from an absolute to a conditional factor, contingent upon the status of modifiable cardiovascular health metrics. This underscores the necessity of integrating comprehensive health assessments like LE8 into the clinical evaluation of musculoskeletal and functional status. Public health and clinical resources aimed at mitigating muscle-related CVD risk should be strategically prioritized for individuals with poor cardiovascular health, particularly those with dysglycemia, sleep disorders, nicotine exposure, obesity, or physical inactivity. Future research should employ longitudinal designs to track the dynamics of LE8 and muscle strength, which will be pivotal for developing precise, integrated preventive strategies.

## Data Availability

The datasets presented in this study can be found in online repositories. The names of the repository/repositories and accession number(s) can be found below: data used in the current study were from the UK Biobank study with application number 79612. Researchers can request the data we used upon approval from the UK Biobank study.
