# Supplementary material for: The synergy between life’s essential 8 and muscle strength on cardiovascular disease risk
Source: Front Med (Lausanne). 2025 Oct 23;12:1628066. doi: 10.3389/fmed.2025.1628066 (PMC12589015; doi:10.3389/fmed.2025.1628066)
Supplement: Supplementary file 1 [file Table_1.pdf]

Supplementary Material

Supplementary Table 1. Tertiles of absolute muscle strength.

Sex	Age	Tertiles 1/Low	Tertiles 2/Medium	Tertiles 3/High
Male	<56 years	<39 kg	39-46 kg	>46 kg
	56-65 years	<36 kg	36-42 kg	>42 kg
	>65 years	<33 kg	33-39 kg	>39 kg
Female	<56 years	<23 kg	23-28 kg	>28 kg
	56-65 years	<20 kg	20-25 kg	>25 kg
	>65 years	<19 kg	19-23 kg	>23 kg

Supplementary Figure 1. Muscle strength distribution in different groups by sex and age.

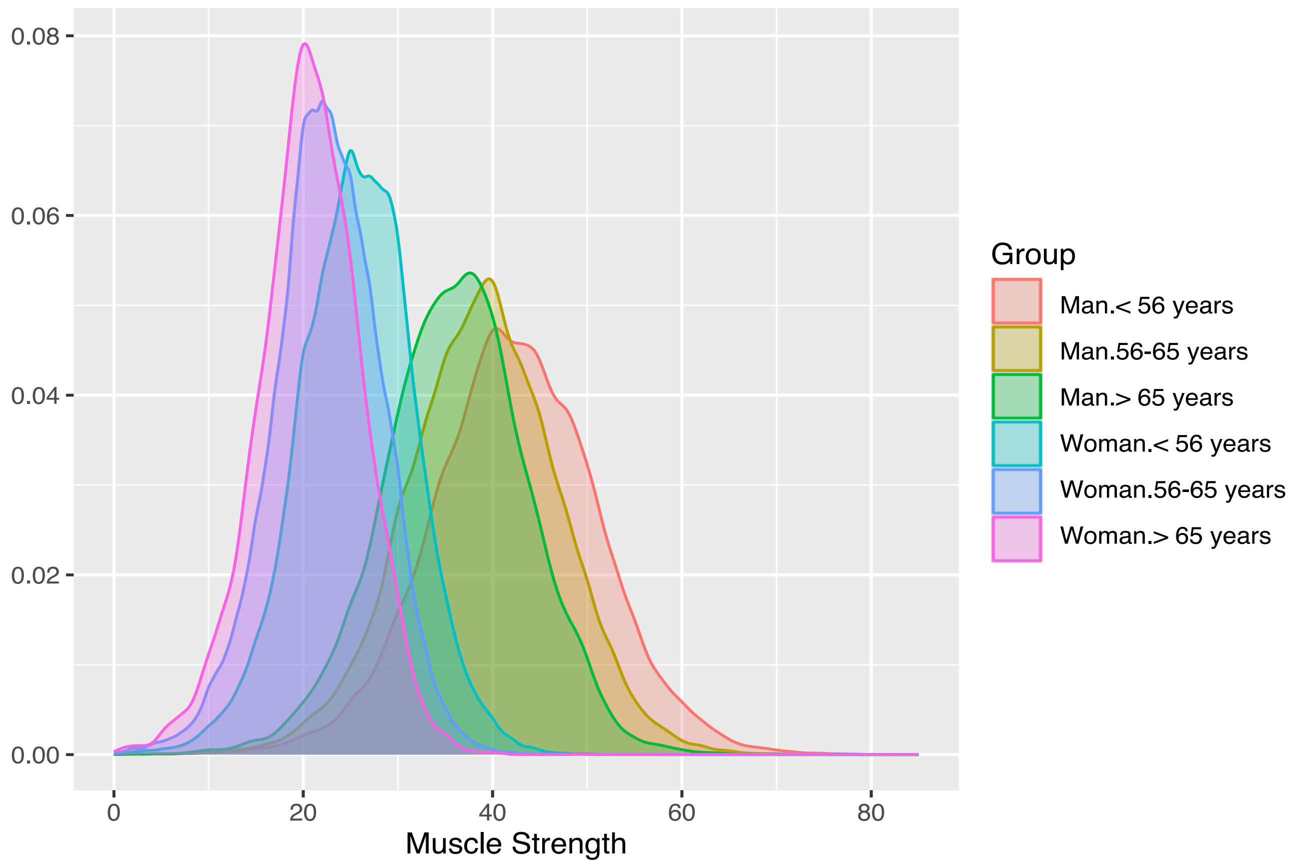

Supplementary Table 2. Assessment scale of life's essential 8.

Health factors	Field IDs	Scoring criteria
Diet	Details in healthy diet score.	≥95th percentile, 100 points;<br>75th–94th percentile, 80 points;<br>50th–74th percentile, 50 points;<br>25th–49th percentile, 25 points;<br>1st–24th percentile, 0 point.
Physical activity	Moderate activity (894)<br>Intensity activity (914)	≥150, 100 points;<br>120 ~ 149.9, 90 points;<br>90 ~ 119.9, 80 points;<br>60 ~ 89.9, 60 points;<br>30 ~ 59.9, 40 points;<br>1 ~ 29.9, 20 points;<br>0, 0 point.
Nicotine exposure	Smoking status (20116)<br>Recruitment age (21022)<br>Stop smoking age (2897)<br>Secondhand (1259)	Never smoker, 100 points;<br>Former smokers with quit years:<br>≥5, 75 points;<br>1~5, 50 points;<br><1, 25 points;<br>Current smoker, 0 point.<br>Subtract 20 of secondhand smoke.

Sleep health	Sleep time (1160)	≥7 and <9, 100 points;<br>≥9 or <10, 90 points;<br>≥6 and <7, 70 points;<br>≥5 and <6/ ≥10, 40 points;<br>≥4 and <5, 20 points;<br><4, 0 point
BMI	BMI (23104)	<25, 100 points;<br>25~29.9, 70 points;<br>30~34.9, 30 points;<br>35~39.9, 15 points;<br>≥40, 0 point.
Blood lipid	Cholesterol (30690)<br>HDL-C (30760)<br>Lipid drug (6177, 6153)	<130, 100 points;<br>130~159, 60 points;<br>160~189, 40 points;<br>190~219, 20 points;<br>≥220, 0 point.<br>If drug-treated, subtract 20 points
Blood glucose	FBG (30740)<br>HbA1c (30750)<br>Diabetes (2443)	No diabetes, FBG <100 or HbA1c <5.7, 100 points;<br>No diabetes, FBG 100-125 (or HbA1c 5.7–6.4), 60 points;<br>Diabetes, HbA1c <7.0, 40 points;<br>Diabetes, HbA1c 7.0–7.9, 30 points;<br>Diabetes, HbA1c 8.0–8.9, 20 points;<br>Diabetes, Hb A1c 9.0–9.9, 10 points;<br>Diabetes, HbA1c ≥10, 0 point.

Blood pressure	SBP (4080)<br><br>DBP (4079)<br><br>HTN drug (6177, 6153)	SBP <120, DBP <80, 100 points;<br><br>120 ≤SBP <130 and DBP <80, 75 points;<br><br>130 ≤SBP <140 or 80 ≤DBP <90, 50 points;<br><br>140 ≤SBP <160 or 90 ≤DBP <100, 25 points;<br><br>SBP ≥160 or DBP ≥100, 0 point.<br><br>Subtract 20 if treated level.

Supplementary Table 3. Healthy Diet Score using touchscreen questionnaire in the UK Biobank study.

Food	IDs	Definition of criterion	Score
Consume more			
Fruits, fresh or dried	1309<br>1319	≥3 servings per day including fresh and dried fruits.<br>1 piece of dried fruit (e.g. apricot) ~=2.5 TBSP, 1 TBSP= 0.063 cups; ½ cup of dried fruit (1 serving) is 3 pieces of dried fruit.<br>1 medium sized fruit is one serving.	1, meet; 0, not meet.
Vegetables, salad or cooked	1289<br>1299	≥3 servings per day including salad, raw and cooked.<br>1 cup of raw leafy vegetables is 16 TBSP. ½ cup of cooked or non-leafy raw vegetables is 8 TBSP.<br>1 serving of raw leafy or non-leafy vegetables is on average ~=12 TBSP; 1 serving of cooked vegetables is ~=8 TBSP.	1, meet; 0, not meet.
Whole grains		≥3 servings per day	1, meet; 0, not meet.
Slices of bread	1438<br>1448	Daily slices of wholemeal or wholegrain bread (servings per day), convert from weekly slices	
Cereal	1458<br>1468	Daily bowls of whole wheat cereal as servings/day (bran cereal, biscuit cereal, oat cereal and muesli), convert from weekly bowls	
Fish shellfish	1329<br>1339	Sum weekly frequencies to obtain total servings/week. ≥2 servings/wk	1, meet; 0, not meet.
Oil fish	…		

Non-oil fish	…		
Dairy products	1408<br><br>1418	Reporting consumption of two milk items and eating cheese once a day to meet the 2-3 servings/day criterion.	1, meet; 0, not meet.
Milk	…		
Cheese	…		
Vegetable oil	2654	Reporting use of olive oil or polyunsaturated/sunflower oil (yes=1, 0=no).	1, meet; 0, not meet.
Consume less			
Refined grains, starches, added sugars†	1438<br><br>1448	Follow a similar coding scheme as for whole grains but select non-whole grains; <1.5 servings per day	1, meet; 0, not meet.
Processed meta	1349	Once a week or less would meet the criterion.	1, meet; 0, not meet.
Unprocessed red meat	1369<br><br>1379<br><br>1389	Summation of frequency of consumption across three types of red meats (lamb/mutton, beef or pork). <3 on the summation corresponds to the criterion of <1-2 servings per week.	1, meet; 0, not meet.
Industrial trans fat	1428	Never use spread, e.g. butter or margarine etc. would meet the criterion	1, meet; 0, not meet.
Sugar-sweetened beverages	6144	Never eat sugar or food/drink containing sugar would meet the criterion	1, meet; 0, not meet.
Sodium	1478	Salt added to food, never or rarely would meet the criterion	1, meet; 0, not meet.

Supplementary Table 4. Cardiovascular Disease Definitions in UK Biobank Study.

Disease	ICD-10	Self-reported field IDs
Coronary heart disease (CHD)	I20, I21, I22, I23, I24, I25	131296, 131297, 131298, 131299, 131300, 131301, 131302, 131303, 131304, 131305, 131306, 131307
Heart failure (HF)	I50	131354, 131355
Stroke	I60, I61, I62, I63, I64	131360, 131361, 131362, 131363, 131364, 131365, 131366, 131367, 131368, 131369

Supplementary Figure 2. Associations of LE8 score with the risk of CVD in Cox models with restricted cubic splines after adjustment.

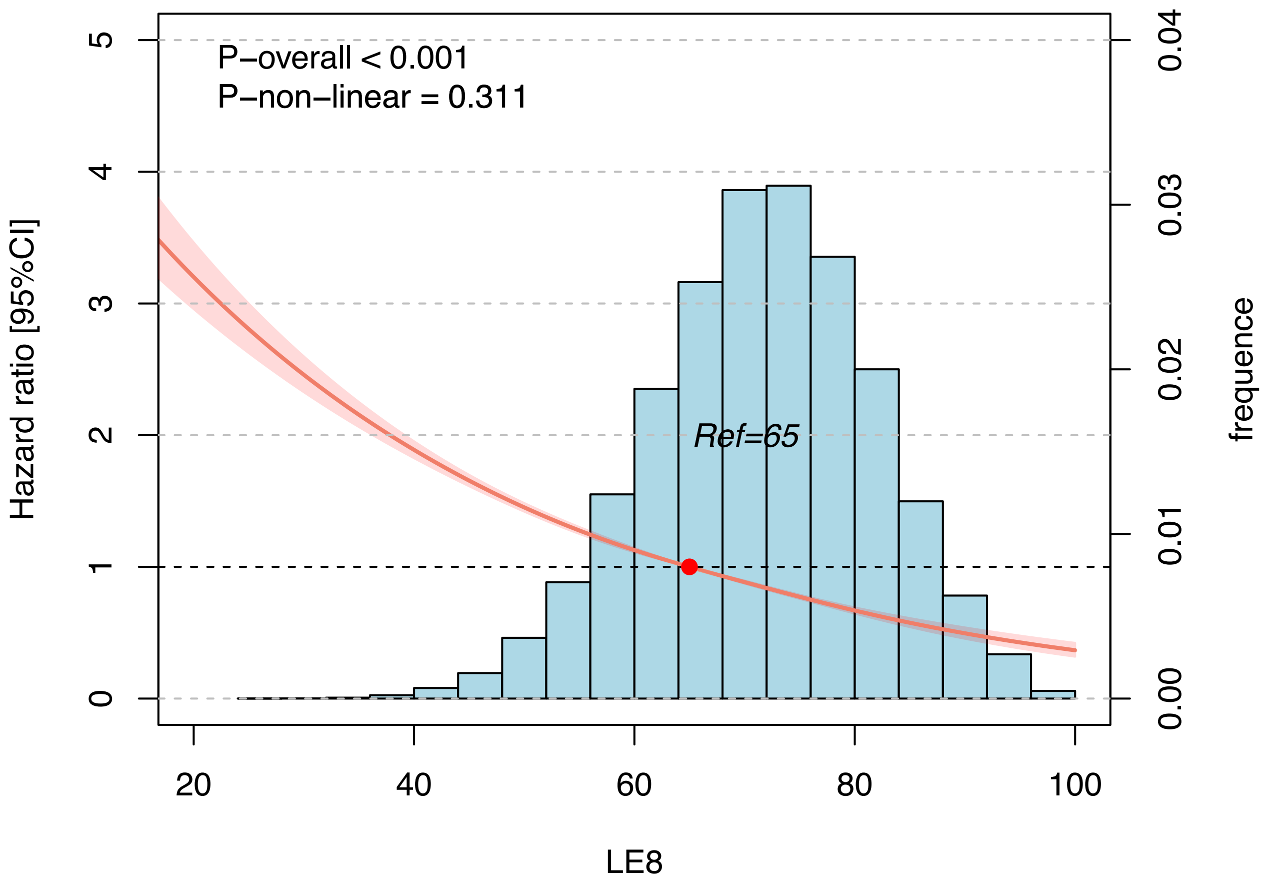

7 **Supplementary Table 5.** Hazard Ratios for Incident CVD per 10-Score LE8 Decrease in Different groups of Muscle Strength. Muscle strength was categorized into three groups-high, medium, and low-based on trichotomies stratified by gender and age. Analysis was adjusted for age, sex, race, height, education, income, alcohol, hyperthyroidism, depression and chronic kidney failure. Abbreviations: LE8, Life's essential 8.

LE8 score	High muscle strength	Medium muscle strength	Low muscle strength
Per 10-score decrease	1.28 (1.26-1.31)	1.30 (1.27-1.32)	1.30 (1.28-1.32)

Supplementary Table 6. Tertiles of relative muscle strength.

Sex	Age	Tertiles 1/Low	Tertiles 2/Medium	Tertiles 3/High
Male	<56 years	<1.40 kg/bmi	1.40-1.71 kg/bmi	>1.71 kg/bmi
	56 to 65 years	<1.28 kg/bmi	1.28-1.57 kg/bmi	>1.57 kg/bmi
	>65 years	<1.19 kg/bmi	1.19-1.47 kg/bmi	>1.47 kg/bmi
Female	<56 years	<0.87 kg/bmi	0.87-1.11 kg/bmi	>1.11 kg/bmi
	56 to 65 years	<0.74 kg/bmi	0.74-0.96 kg/bmi	>0.96 kg/bmi
	>65 years	<0.68 kg/bmi	0.68-0.88 kg/bmi	>0.88 kg/bmi

Supplementary Table 7. Joint and interactive association of relative muscle strength and LE8 with CVD incidence. LE8 was categorized as low (LE8 score <50), medium (LE8 score 50-79), or ideal (LE8 score ≥80) based on AHA guidelines. Muscle strength was categorized into three groups-high, medium, and low-based on trichotomies stratified by gender and age. Analysis was adjusted for age, sex, race, education, income, alcohol, hyperthyroidism, depression and chronic kidney failure. Abbreviations: LE8, Life's essential 8; RERI, relative excess risk due to interaction; AP, attributable proportion; SI, synergy index.

	Muscle strength	HR	Multiplicative interaction	Additive interaction	
				RERI	AP
Ideal	High	1 (Reference)	-	-	-
	Medium	1.00 (0.82-1.20)	-	-	-
	Low	1.09 (0.97-1.22)	-	-	-
Medium	High	1.50 (1.39-1.63)	-	-	-
	Medium	1.70 (1.55-1.86)	1.13 (0.93-1.38)	0.20 (-0.004-0.40)	0.12 (-0.004-0.24)
	Low	1.91 (1.77-2.07)	1.17 (1.04-1.32)	0.32 (0.20-0.44)	0.17 (0.10-0.24)
Low	High	2.39 (2.15-2.66)	-	-	-
	Medium	2.84 (2.51-3.21)	1.19 (0.95-1.49)	0.45 (0.09-0.82)	0.16 (0.04-0.28)
	Low	3.07 (2.82-3.34)	1.18 (1.02-1.36)	0.59 (0.37-0.81)	0.19 (0.12-0.27)

10 **Supplementary Table 8.** Joint and interactive association of muscle strength and LE8 with CVD incidence after excluding outcome events in the first three years. LE8 was categorized as low (LE8 score <50), medium (LE8 score 50-79), or ideal (LE8 score ≥80) based on AHA guidelines. Muscle strength was categorized into three groups-high, medium, and low-based on trichotomies stratified by gender and age. Analysis was adjusted for age, sex, race, height, education, income, alcohol, hyperthyroidism, depression and chronic kidney failure. Abbreviations: LE8, Life's essential 8; RERI, relative excess risk due to interaction; AP, attributable proportion; SI, synergy index.

LE8	Muscle strength	HR	Multiplicative interaction	Additive interaction	
				RERI	AP
Ideal	High	1 (Reference)	-	-	-
	Medium	0.88 (0.76-1.02)	-	-	-
	Low	1.01 (0.87-1.16)	-	-	-
Medium	High	1.54 (1.38-1.72)	-	-	-
	Medium	1.60 (1.44-1.78)	1.18 (1.01-1.38)	0.18 (0.04-0.32)	0.11 (0.02-0.21)
	Low	1.73 (1.55-1.93)	1.12 (0.96-1.29)	0.18 (0.04-0.33)	0.11 (0.02-0.19)
Low	High	2.47 (2.19-2.78)	-	-	-
	Medium	2.61 (2.32-2.93)	1.20 (1.01-1.42)	0.25 (0.02-0.49)	0.10 (0.01-0.19)
	Low	2.93 (2.62-3.29)	1.18 (1.01-1.38)	0.46 (0.24-0.68)	0.16 (0.08-0.23)

11 **Supplementary Table 9.** Joint Associations of LE8 and Muscle Strength with CVD: Cause-Specific Hazard Ratios (95% CI). LE8 was categorized as low (LE8 score <50), medium (LE8 score 50-79), or ideal (LE8 score ≥80) based on AHA guidelines. Muscle strength was categorized into three groups-high, medium, and low-based on trichotomies stratified by gender and age. Analysis was adjusted for age, sex, race, height, education, income, alcohol, hyperthyroidism, depression and chronic kidney failure. Abbreviations: LE8, Life's essential 8.

	High Muscle Strength	Medium Muscle Strength	Low Muscle Strength
Ideal LE8	1 (Reference)	0.92 (0.80-1.06)	1.04 (0.91-1.18)
Medium LE8	1.56 (1.41-1.73)	1.61 (1.46-1.79)	1.75 (1.58-1.93)
Low LE8	2.43 (2.18-2.72)	2.64 (2.37-2.95)	2.96 (2.66-3.29

12 **Supplementary Table 10.** Joint and interaction association of muscle strength and LE8 with CVD incidence stratified by sex. LE8 was categorized as low (LE8 score <50), medium (LE8 score 50-79), or ideal (LE8 score ≥80) based on AHA guidelines. Muscle strength was categorized into three groups-high, medium, and low-based on trichotomies stratified by gender and age. Analysis was adjusted for age, sex, race, height, education, income, alcohol, hyperthyroidism, depression and chronic kidney failure. Abbreviations: LE8, Life's essential 8.

LE8	Muscle strength	HR	Multiplicative interaction	Additive interaction	
				RERI	AP
Man					
Ideal	High	1 (Reference)	-	-	-
	Medium	0.87 (0.72-1.05)	-	-	-
	Low	0.92 (0.77-1.10)	-	-	-
Medium	High	1.37 (1.20-1.57)	-	-	-
	Medium	1.43 (1.25-1.64)	1.20 (0.99-1.46)	0.19 (0.02-0.36)	0.13 (0.003-0.26)
	Low	1.51 (1.32-1.73)	1.20 (1.00-1.44)	0.22 (0.06-0.38)	0.14 (0.02-0.26)
Low	High	2.11 (1.82-2.44)	-	-	-
	Medium	2.23 (1.93-2.58)	1.21 (0.98-1.49)	0.25 (-0.002-0.49)	0.11 (-0.004-0.22)
	Low	2.54 (2.21-2.93)	1.30 (1.07-1.59)	0.51 (0.28-0.73)	0.20 (0.10-0.29)
Woman					
Ideal	High	1 (Reference)	-	-	-
	Medium	0.96 (0.78-1.17)	-	-	-
	Low	1.12 (0.92-1.37)	-	-	-
Medium	High	1.77 (1.52-2.06)	-	-	-
	Medium	1.79 (1.54-2.09)	1.06 (0.86-1.32)	0.07 (-0.15-0.29)	0.04 (-0.09-0.17)
	Low	2.02 (1.73-2.35)	1.02 (0.83-1.25)	0.13 (-0.10-0.35)	0.06 (-0.05-0.18)
Low	High	2.96 (2.49-3.52)	-	-	-
	Medium	3.38 (2.85-4.00)	1.20 (0.94-1.52)	0.46 (0.04-0.89)	0.14 (0.01-0.26)
	Low	3.68 (3.12-4.33)	1,11 (0.88-1.39)	0.60 (0.20-1.00)	0.16 (0.05-0.27)
Heterogeneity p-values of sex: P <0.001					

Supplementary Table 11. Joint and interaction association of muscle strength and LE8 with CVD incidence stratified by age. LE8 was categorized as low (LE8 score <50), medium (LE8 score 50-79), or ideal (LE8 score ≥80) based on AHA guidelines. Muscle strength was categorized into three groups-high, medium, and low-based on trichotomies stratified by gender and age. Analysis was adjusted for age, sex, race, height, education, income, alcohol, hyperthyroidism, depression and chronic kidney failure. Abbreviations: LE8, Life’s essential 8.

LE8	Muscle strength	HR	Multiplicative interaction	Additive interaction	
				RERI	AP
< 56 years					
Ideal	High	1 (Reference)	-	-	-
	Medium	0.84 (0.65-1.09)	-	-	-
	Low	1.11 (0.88-1.41)	-	-	-
Medium	High	1.96 (1.63-2.36)	-	-	-
	Medium	2.06 (1.72-2.48)	1.25 (0.95-1.63)	0.26 (0.005-0.51)	0.13 (-0.008-0.26)
	Low	2.29 (1.91-2.76)	1.05 (0.82-1.35)	0.22 (-0.05-0.49)	0.10 (-0.03-0.22)
Low	High	3.61 (2.94-4.42)	-	-	-
	Medium	4.03 (3.29-4.93)	1.32 (0.99-1.77)	0.58 (0.02-1.14)	0.14 (0.01-0.28)
	Low	4.87 (4.01-5.91)	1.21 (0.93-1.59)	1.15 (0.61-1.69)	0.24 (0.13-0.34)
56-65 years					
Ideal	High	1 (Reference)	-	-	-
	Medium	1.03 (0.84-1.26)	-	-	-
	Low	1.16 (0.95-1.40)	-	-	-
Medium	High	1.57 (1.36-1.83)	-	-	-
	Medium	1.70 (1.47-1.98)	1.05 (0.86-1.30)	0.10 (-0.11-0.31)	0.06 (-0.07-0.19)
	Low	1.86 (1.60-2.15)	1.02 (0.84-1.24)	0.13 (-0.08-0.34)	0.07 (-0.05-0.19)
Low	High	2.45 (2.09-2.89)	-	-	-
	Medium	2.72 (2.32-3.20)	1.08 (0.86-1.35)	0.24 (-0.08-0.56)	0.09 (-0.03-0.21)
	Low	3.05 (2.61-3.56)	1.07 (0.87-1.33)	0.44 (0.14-0.74)	0.14 (0.04-0.25)
> 65years					
	High	1 (Reference)	-	-	-

Ideal	Medium	0.87 (0.65-1.18)	-	-	-
	Low	0.85 (0.64-1.14)	-	-	-
Medium	High	1.20 (0.97-1.48)	-	-	-
	Medium	1.24 (1.002-1.53)	1.18 (0.87-1.61)	0.17 (0.10-0.44)	0.13 (-0.10-0.37)
	Low	1.36 (1.10-1.68)	1.33 (0.99-1.80)	0.31 (0.07-0.55)	0.23 (0.02-0.43)
Low	High	1.68 (1.33-2.12)	-	-	-
	Medium	1.89 (1.50-2.39)	1.29 (0.92-1.81)	0.34 (-0.02-0.70)	0.18 (-0.02-0.38)
	Low	2.00 (1.60-2.51)	1.40 (1.01-1.94)	0.47 (0.13-0.81)	0.23 (0.060 0.41)
Heterogeneity p-values of age: P <0.001					

14 **Supplementary Table 12.** Joint and interaction association of muscle strength and LE8 with CVD incidence stratified by disease subtypes. LE8 was categorized as low (LE8 score <50), medium (LE8 score 50-79), or ideal (LE8 score ≥80) based on AHA guidelines. Muscle strength was categorized into three groups-high, medium, and low-based on trichotomies stratified by gender and age. Analysis was adjusted for age, sex, race, height, education, income, alcohol, hyperthyroidism, depression and chronic kidney failure. Abbreviations: LE8, Life's essential 8; HF, heart failure; CHD, coronary heart disease.

LE8	Muscle strength	HR	Multiplicative interaction	Additive interaction	
				RERI	AP
HF					
Ideal	High	1 (Reference)	-	-	-
	Medium	0.98 (0.71-1.34)	-	-	-
	Low	1.38 (1.04-1.84)	-	-	-
Medium	High	1.70 (1.35-2.14)	-	-	-
	Medium	1.79 (1.42-2.25)	1.08 (0.78-1.49)	0.11 (-0.21-0.43)	0.06 (-0.13-0.25)
	Low	2.09 (1.66-2.63)	0.89 (0.66-1.19)	0.0004 (-0.36-0.36)	0.0002 (-0.17- 0.17)
Low	High	3.16 (2.46-4.04)	-	-	-
	Medium	3.42 (2.67-4.37)	1.11 (0.79-1.57	0.29 (-0.24- 0.81)	0.08 (-0.07-0.24)
	Low	4.43 (3.49-5.61)	1.01 (0.74-1.38)	0.89 (0.37-1.40)	0.20 (0.08- 0.32)
CHD					
Ideal	High	1 (Reference)	-	-	-
	Medium	0.93 (0.78-1.11)	-	-	-
	Low	1.09 (0.93-1.29)	-	-	-
Medium	High	1.77 (1.56-2.00)	-	-	-
	Medium	1.83 (1.61-2.07)	1.11 (0.93-1.33)	0.13 (-0.04-0.30)	0.07 (-0.03- 0.17)
	Low	1.93 (1.70-2.19)	1.00 (0.85-1.18)	0.07 (-0.11-0.25)	0.04 (-0.06-0.13)
Low	High	2.85 (2.48-3.26)	-	-	-
	Medium	3.00 (2.62-3.44)	1.13 (0.94-1.37)	0.23 (-0.06-0.51)	0.08 (-0.02-0.17)
	Low	3.35 (2.94-3.82)	1.08 (0.90-1.29)	0.41 (0.14-0.68)	0.12 (0.04- 0.21)
Stroke					
	High	1 (Reference)	-	-	-

Ideal	Medium	0.82 (0.63-1.08)	-	-	-
	Low	0.81 (0.62-1.06)	-	-	-
Medium	High	1.24 (1.02-1.51)	-	-	-
	Medium	1.26 (1.03-1.53)	1.23 (0.92-1.63)	0.19 (-0.05-0.43)	0.15 (-0.06-0.36)
	Low	1.39 (1.14-1.69)	1.38 (1.05-1.83)	0.34 (0.12- 0.56)	0.24 (0.06- 0.43)
Low	High	1.76 (1.41-2.20)	-	-	-
	Medium	1.90 (1.53-2.37)	1.31 (0.96-1.80)	0.32 (-0.04-0.68)	0.17 (-0.03-0.36)
	Low	2.09 (1.69-2.58)	1.47 (1.08-1.99)	0.52 (0.19-0.85)	0.25 (0.08-0.42)
Heterogeneity p-values of disease subtypes: P <0.001					

Supplementary Table 13. Joint and interaction association of muscle strength and LE8 with HF incidence after excluding outcome events in the first three years. LE8 was categorized as low (LE8 score <50), medium (LE8 score 50-79), or ideal (LE8 score ≥80) based on AHA guidelines. Muscle strength was categorized into three groups-high, medium, and low-based on trichotomies stratified by gender and age. Analysis was adjusted for age, sex, race, height, education, income, alcohol, hyperthyroidism, depression and chronic kidney failure. Abbreviations: LE8, Life's essential 8.

LE8	Muscle strength	HR	Multiplicative interaction	Additive interaction	
				RERI	AP
Ideal	High	1 (Reference)	-	-	-
	Medium	1.11 (0.81-1.52)	-	-	-
	Low	1.31 (0.97-1.78)	-	-	-
Medium	High	1.72 (1.35-2.19)	-	-	-
	Medium	1.83 (1.44-2.33)	0.96 (0.69-1.33)	0.003 (-0.36-0.36)	0.002 (-0.19-0.20)
	Low	2.12 (1.67-2.70)	0.94 (0.68-1.28)	0.08 (-0.28-0.44)	0.04 (-0.14-0.21)
Low	High	3.20 (2.47-4.14)	-	-	-
	Medium	3.42 (2.65-4.41)	0.97 (0.68-1.37)	0.11 (-0.46-0.68)	0.03 (-0.13-0.20)
	Low	4.39 (3.43-5.63)	1.04 (0.75-1.45)	0.88 (0.34-1.42)	0.20 (0.07-0.33)
